# Fetal and Long-Term Maternal Outcomes of New-Onset Arrhythmia During Pregnancy: An Epic Cosmos Analysis

**DOI:** 10.1016/j.jacadv.2026.103135

**Published:** 2026-08-20

**Authors:** Jingwen Huang, Sheida Habibi, Benjamin W. Furman, Anna Miele, Ijeoma Eleazu, Gabriel R. Najarro, Neal K. Bhatia, Mikhael F. El-Chami, Faisal M. Merchant

**Affiliations:** a Division of Cardiology, Department of Medicine, Emory University School of Medicine, Atlanta, Georgia, USA; b Department of Biomedical Informatics, Emory University School of Medicine, Atlanta, Georgia, USA; c Emory Clinical Cardiovascular Research Institute, Division of Cardiology, Emory University School of Medicine, Atlanta, Georgia, USA

**Keywords:** atrial fibrillation, atrial flutter, cardiovascular outcomes, fetal outcomes, pregnancy, supraventricular tachycardia

## Abstract

**Background:**

Physiologic changes during pregnancy can trigger arrhythmias such as atrial fibrillation/flutter (AF/AFL) and supraventricular tachycardia (SVT), which are associated with short-term maternal and fetal morbidity. Whether these arrhythmias represent a transient risk or are associated with long-term risk is not well characterized.

**Objectives:**

The purpose of this study was to examine long-term maternal cardiovascular and fetal outcomes among pregnant women with new-onset vs pre-existing AF/AFL or SVT compared with arrhythmia-free controls, using the Epic Cosmos database.

**Methods:**

In AF/AFL and SVT cohorts, we compared women with new-onset arrhythmia during pregnancy (AF/AFL N = 2,978; SVT N = 12,860) to women with pre-existing arrhythmia (AF/AFL N = 2,996; SVT N = 45,288) and arrhythmia-free women (AF/AFL N = 7,676,692; SVT N = 6,553,510). Long-term maternal major adverse cardiovascular events (MACE) were analyzed using multivariable Cox proportional hazards models and fetal outcomes using logistic regressions.

**Results:**

Compared with arrhythmia-free controls, both new-onset and pre-existing AF/AFL were associated with increased long-term MACE risk (HR: 3.18 and 3.02, both *P* < 0.001), with similar risk between groups. New-onset and pre-existing SVT were associated with increased MACE risk (HR: 1.96 and 3.83, both *P* < 0.001), with pre-existing SVT carrying higher risk than new-onset SVT (HR: 1.95, *P* < 0.001). Both new-onset and pre-existing AF/AFL were associated with worse fetal outcomes, including preterm birth and stillbirth, while new-onset SVT had higher preterm birth risk than pre-existing SVT.

**Conclusions:**

New-onset AF/AFL and SVT during pregnancy were associated with increased long-term maternal MACE risk, extending well beyond the peripartum period. A first diagnosis of AF/AFL or SVT during pregnancy may identify women at elevated long-term cardiovascular risk warranting structured follow-up.

Cardiac arrhythmias are increasingly prevalent during pregnancy, in part driven by an increase in the average age of childbearing.^1^^,^^2^ The hemodynamic adaptations of pregnancy, including increased plasma volume, elevated resting heart rate, and shifts in autonomic tone,^3^^,^^4^ create an electrophysiological stress which may trigger de novo arrhythmias in pregnant women without any prior arrhythmic history. Atrial fibrillation (AF) and atrial flutter (AFL) and supraventricular tachycardia (SVT) are the dominant sustained arrhythmias encountered during pregnancy.^5^^,^^6^ The need for hospitalization for arrhythmia during pregnancy is associated with an increased risk of both maternal and fetal complications.^7^^,^^8^ In a 10-year retrospective analysis of maternal cardiovascular deaths, arrhythmias were identified as the immediate or underlying cause in 10.7% of cases.^9^

Prior studies on new-onset arrhythmias during pregnancy have been limited by small cohort sizes and short-term follow-up, primarily confined to the peripartum period.^2^^,^^10^ The long-term prognostic significance of new-onset arrhythmias during pregnancy remains poorly characterized. A clinically important unresolved question is whether new-onset arrhythmia during pregnancy represents a transient, reversible risk or whether it serves as a marker of an underlying electrophysiologic and structural substrate which is associated with long-term adverse cardiovascular risk. Answering this question has important implications for postpartum risk stratification, surveillance, and patient counseling.

To address this knowledge gap, we leveraged the Epic Cosmos database—a nationwide network encompassing over 300 million patients—to examine long-term maternal clinical outcomes and fetal outcomes in pregnant women with new-onset AF/AFL or SVT.

## Methods

### Data source

This retrospective cohort study was conducted using de-identified electronic health record (EHR) data from the Cosmos database derived from the Epic health system, which aggregates clinical data from multiple participating healthcare institutions. The study included records from February 2000 through December 2024. All patients in the United States who had a pregnancy during this period and had available demographic information in the database were eligible for inclusion. At the time of this study, the Cosmos community included data from approximately 300 million patients, spanning 351 organizations, 1,989 hospitals, and 44,800 clinics across 4 countries, providing broad geographic and clinical representation. The use of de-identified data qualified for a waiver of institutional review board approval and need for informed consent. The AF/AFL and SVT analyses were conducted using separate data extractions; differences in cohort sizes between the 2 analyses reflect the dynamic nature of the Epic Cosmos database, where data availability may change over time due to ongoing deidentification and data governance updates.

### Study population

**Figure 1:**
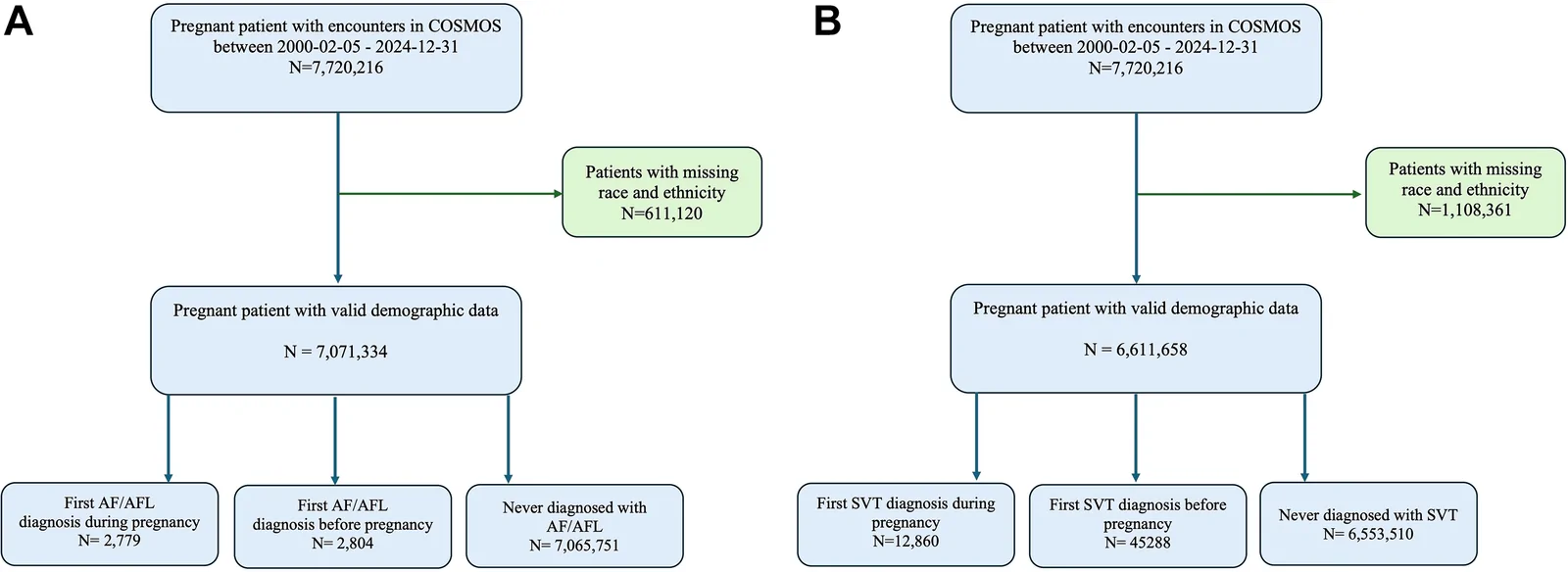
Flowchart Flowchart of study population selection from the Epic Cosmos database for the AF/AFL cohort (A) and the SVT cohort (B), detailing exclusions for missing race and ethnicity data and final allocation into new-onset, pre-existing, and arrhythmia-free groups. Differences in missingness between the 2 cohorts likely reflect the temporal gap between data extractions, as Epic Cosmos data availability can change over time due to ongoing deidentification, masking, or data governance updates applied to the database. AF = atrial fibrillation; AFL = atrial flutter; SVT = supraventricular tachycardia.

Patients with documented pregnancies were categorized according to the presence and timing of AF/AFL and SVT diagnoses relative to pregnancy. For both arrhythmias, patients were stratified into 3 mutually exclusive groups: those with a first diagnosis occurring during pregnancy, those with a first diagnosis prior to pregnancy but not during pregnancy, and those with no recorded history of the arrhythmia (Central Illustration). For patients whose first diagnosis occurred during pregnancy, that pregnancy was defined as the index pregnancy. For patients with a first diagnosis prior to pregnancy but without a diagnosis during pregnancy, the first pregnancy following the diagnosis was considered the index pregnancy. For patients with no recorded history of the arrhythmia, the first pregnancy during the study period was used as the index pregnancy. The AF/AFL and SVT analyses were conducted using separate data extractions; differences in cohort sizes between the 2 analyses reflect the dynamic nature of the Epic Cosmos database, where data availability may change over time because of ongoing deidentification and data governance updates. Patients with missing race and/or ethnicity data were excluded from all analyses (Figure 1). For pregnancies with a missing gestational end date, gestational duration was imputed as 9 months from the pregnancy start date. Diagnosis and procedure codes (ICD-10/Current Procedural Terminology [CPT]) were treated as present or absent based on coded clinical records.

**Central Illustration:**
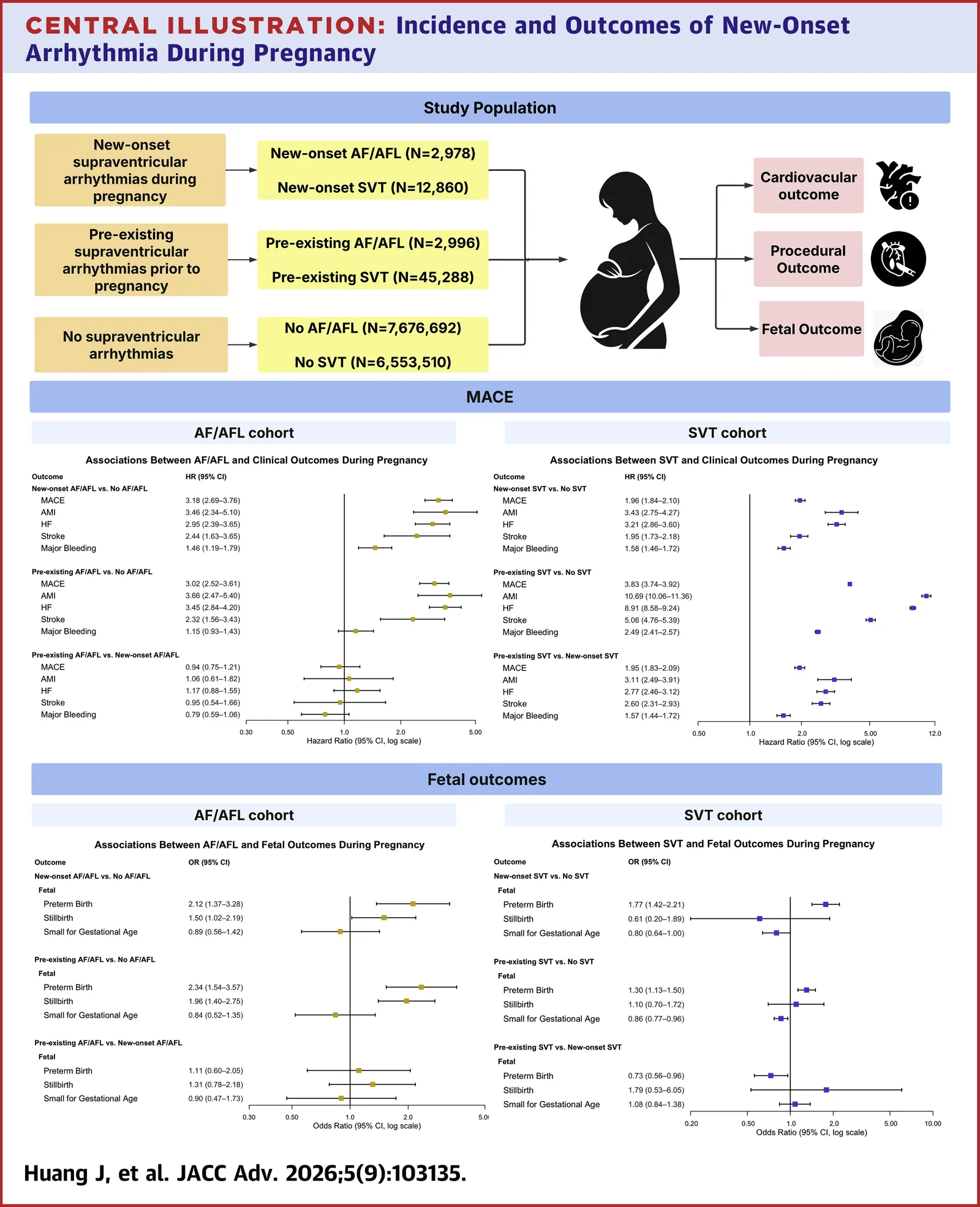
Incidence and Outcomes of New-Onset Arrhythmia During Pregnancy Schematic overview of the study design and key findings: pregnant women were stratified by supraventricular arrhythmia status (new-onset during pregnancy, pre-existing, or none) and evaluated for long-term clinical, procedural, and fetal outcomes, with forest plots depicting adjusted HRs or odds ratios—with corresponding HR/OR (95% CI) values displayed—for clinical and fetal outcomes across both the AF/AFL and SVT cohorts. AF = atrial fibrillation; AFL = atrial flutter; MACE = major adverse cardiovascular events; SVT = supraventricular tachycardia.

### Study outcomes

Clinical outcomes were identified using the International Classification of Diseases–10th Revision–Clinical Modification (ICD-10-CM) codes recorded in the EHR. The primary outcome was the incidence of major adverse cardiovascular events (MACE), defined as the composite incidence of acute myocardial infarction (AMI), stroke, and heart failure (HF) hospitalization. Secondary outcomes were categorized into clinical outcomes, procedural outcomes, and fetal outcomes. Clinical outcomes include the incidence of AMI, HF hospitalization, stroke, and major bleeding (a composite of gastrointestinal bleeding and intracranial bleeding). Procedural outcomes include the need for cardioversion, catheter ablation, and pacemaker or defibrillator implantation. Fetal outcomes included preterm birth, small for gestational age (SGA), and stillbirth at the time of delivery. Procedural outcomes were identified using CPT codes for outpatient encounters and ICD-10, Procedure Coding System codes for inpatient encounters. Fetal outcomes were also identified using ICD-10-CM codes. Preterm birth and SGA derived from newborn diagnoses were linked back to the maternal EHR. Stillbirth was based on the diagnosis in the maternal EHR. A complete list of ICD-10 and CPT codes for covariate identification is provided in Supplemental Table 1, and that for outcomes identification is shown in Supplemental Table 2.

### Statistical analysis

Descriptive statistics were used to summarize baseline characteristics across arrhythmia groups. Categorical variables are reported as counts and percentages, while continuous variables are summarized using means and standard deviations. Time-to-event analyses were performed to evaluate the association between arrhythmia status and outcomes. Cox proportional hazards regression models were used to estimate HRs and CIs for the association between AF/AFL or SVT status and outcomes. The proportional hazards assumption was assessed using Schoenfeld residuals, and given the large study population, a conservative threshold of a value of *P* < 0.01 was used to indicate violation of the assumption and guide stratification in the Cox models. Time-to-event was defined as the interval between the index pregnancy and the first occurrence of an outcome of interest, with individuals who did not experience the outcome censored at the end of the study period. Cox models were adjusted for demographic characteristics, cardiovascular comorbidities, and pregnancy-related complications. Demographic variables included maternal age and race/ethnicity. Clinical comorbidities included hypertension (HTN), diabetes mellitus, valvular heart disease, and prior cardiac device implantation. Pregnancy-related covariates included gestational HTN, preeclampsia or eclampsia, gestational diabetes, preterm labor, anemia during pregnancy, obesity, and tobacco or alcohol use during pregnancy. For fetal outcomes, multivariable logistic regression models were applied to evaluate differences in the odds of adverse pregnancy outcomes between arrhythmia groups. For multivariable logistic regression models, only age, race, gestational HTN, gestational diabetes, and pre-eclampsia were adjusted to avoid model overfitting. Statistical analyses were conducted using R Software (version 4.5.1) located in Epic Cosmos Data Science Virtual Machine, and significance was assessed using two-sided tests.

## Results

### Atrial fibrillation/flutter cohort

Out of 7,682,666 women with pregnancies, we identified 2,978 (0.039%) women with a new diagnosis of AF/AFL during pregnancy and 2,996 (0.039%) women with a pre-existing diagnosis of AF/AFL. Median follow-up duration after the index pregnancy in the AF/AFL cohort was 6.6 years (interquartile range: 3.7 to 9.7). Baseline characteristics stratified by AF/AFL status are presented in Table 1. In general, women with new-onset AF/AFL during pregnancy tended to represent an intermediate phenotype. Women with new-onset AF/AFL during pregnancy tended to be older and have a greater burden of comorbidities than pregnant women without any history of AF/AFL. However, women with new-onset AF/AFL during pregnancy tended to be younger and have fewer comorbidities than pregnant women with a pre-existing diagnosis of AF/AFL.

**Table 1:** Baseline Characteristics of the AF/AFL Cohort

	New-Onset AF/AFL (n = 2,978)	Pre-Existing AF/AFL (n = 2,996)	No AF/AFL (n = 7,676,692)	*P* Value
Age, y				<0.0001
<20	102 (3.7%)	52 (1.9%)	518,519 (7.3%)	
20-29	1,291 (46.5%)	1,208 (43.1%)	3,587,288 (50.8%)	
30-34	799 (28.8%)	849 (30.3%)	1,892,185 (26.8%)	
35-39	458 (16.5%)	555 (19.8%)	873,108 (12.4%)	
≥40	129 (4.6%)	140 (5.0%)	194,651 (2.8%)	
Race/ethnicity				<0.0001
Hispanic	345 (11.6%)	291 (9.7%)	1,529,608 (19.9%)	
American Indian/Alaska Native	51 (1.7%)	45 (1.5%)	116,281 (1.5%)	
Asian	102 (3.4%)	88 (2.9%)	469,260 (6.1%)	
Black	744 (25.0%)	621 (20.7%)	1,343,549 (17.5%)	
Native Hawaiian/Pacific Islander	14 (0.5%)	20 (0.7%)	49,144 (0.6%)	
White	1,839 (61.8%)	2,045 (68.3%)	4,727,019 (61.6%)	
Comorbidities				
Type 2 diabetes mellitus	20 (0.7%)	102 (3.4%)	23,125 (0.3%)	<0.0001
Hypertension	27 (0.9%)	185 (6.2%)	45,877 (0.6%)	<0.0001
Device implant	31 (1.0%)	40 (1.3%)	636 (0.0%)	<0.0001
Valvular heart disease	344 (11.6%)	479 (16.0%)	51,947 (0.7%)	<0.0001
Pregnancy-related comorbidities				
Gestational hypertension	190 (6.4%)	286 (9.5%)	245,832 (3.2%)	<0.0001
Preeclampsia and/or eclampsia	29 (1.0%)	90 (3.0%)	67,954 (0.9%)	<0.0001
Gestational diabetes	190 (6.4%)	232 (7.7%)	349,683 (4.6%)	<0.0001
Preterm labor	402 (13.5%)	331 (11.0%)	431,059 (5.6%)	<0.0001
Anemia during pregnancy	621 (20.9%)	522 (17.4%)	642,714 (8.4%)	<0.0001
Obesity during pregnancy	674 (22.6%)	652 (21.8%)	814,694 (10.6%)	<0.0001
Smoking during pregnancy	325 (10.9%)	412 (13.8%)	373,957 (4.9%)	<0.0001
Alcohol abuse	9 (0.3%)	24 (0.8%)	8,785 (0.1%)	<0.0001

Values are n (%) and median (Q1-Q3), unless otherwise indicated.

AF = atrial fibrillation; AFL = atrial flutter.

#### Long-term maternal outcomes

**Figure 2:**
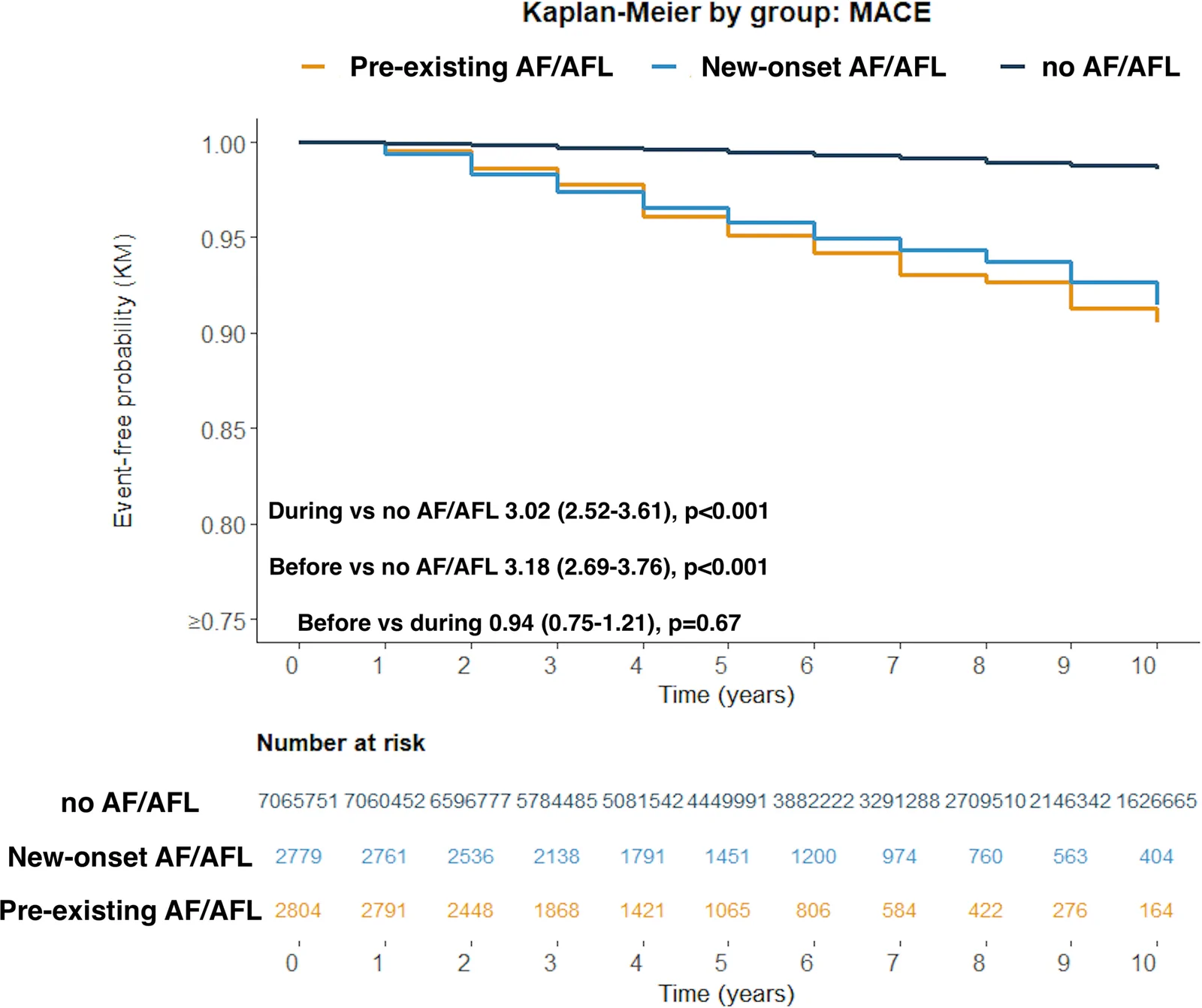
Kaplan-Meier Curves for MACE-Free Probability of AF/AFL Cohort Kaplan-Meier curves for women with new-onset AF/AFL during pregnancy (n = 2,978), pre-existing AF/AFL prior to pregnancy (n = 2,996), and no AF/AFL history (n = 7,676,692). Event-free probability of MACE is plotted from the index pregnancy encounter over 10 years of follow-up. Numbers at risk are shown below each panel at yearly intervals. AF = atrial fibrillation; AFL = atrial flutter; MACE = major adverse cardiovascular events.

Both AF/AFL groups experienced substantially increased long-term cardiovascular risk compared with arrhythmia-free controls (Table 2, Figure 2). Women with new-onset AF/AFL during pregnancy had a significantly increased risk of MACE (HR: 3.18, 95% CI: 2.69-3.76), as well as an increased risk of AMI (HR: 3.46; 95% CI: 2.34-5.10), HF hospitalization (HR: 2.95; 95% CI: 2.39-3.65), stroke (HR: 2.44; 95% CI: 1.63-3.65), and major bleeding (HR: 1.46; 95% CI: 1.19-1.79) (all *P* < 0.001 compared to arrhythmia-free controls). Similarly, pregnant women with a pre-existing diagnosis of AF/AFL also had significantly increased long-term risk compared to arrhythmia-free controls for MACE, AMI, HF hospitalization, and stroke (HR: 3.02, 3.66, 3.45, and 2.32, respectively, all *P* < 0.001). The incidence of long-term adverse outcomes in pregnant women with new-onset AF/AFL was similar to that in those with pre-existing AF/AFL. In a sensitivity analysis excluding HF hospitalization from the MACE definition (composite of AMI and stroke only), associations remained significant for both new-onset AF/AFL (HR: 2.88; 95% CI: 2.18-3.80; *P* < 0.001) and pre-existing AF/AFL (HR: 3.09; 95% CI: 2.34-4.10; *P* < 0.001) compared to controls, with similar risk between the 2 AF/AFL groups (HR: 1.08; *P* = 0.716) (Supplemental Table 3).

**Table 2:** Event Rates and Outcomes for AF/AFL Cohort

	Event Rates			Outcomes		
	New-Onset AF/AFL	Pre-Existing AF/AFL	No AF/AFL	New-Onset AF/AFL vs No AF/AFL HR/OR (95% CI), *P* Value	Pre-Existing AF/AFL vs No AF/AFL HR/OR (95% CI), *P* Value	Pre-Existing AF/AFL vs New-Onset AF/AFL HR/OR (95% CI), *P* Value
Clinical outcomes						
MACE	142 (5.11%)	126 (4.49%)	66,523 (0.94%)	3.18 (2.69-3.76), *P* < 0.001	3.02 (2.52-3.61), *P* < 0.001	0.94 (0.75-1.21), *P* = 0.67
AMI	26 (0.94%)	26 (0.93%)	12,066 (0.17%)	3.46 (2.34-5.10), *P* < 0.001	3.66 (2.47-5.40), *P* < 0.001	1.06 (0.61-1.82), *P* = 0.84
HF	91 (3.25%)	106 (3.81%)	39,445 (0.56%)	2.95 (2.39-3.65), *P* < 0.001	3.45 (2.84-4.20), *P* < 0.001	1.17 (0.88-1.55), *P* = 0.28
Stroke	24 (0.86%)	25 (0.90%)	20,858 (0.30%)	2.44 (1.63-3.65), *P* < 0.001	2.32 (1.56-3.43), *P* < 0.001	0.95 (0.54-1.66), *P* = 0.86
Major bleeding	92 (3.28%)	85 (3.56%)	178,412 (2.53%)	1.46 (1.19-1.79), *P* < 0.001	1.15 (0.93-1.43), *P* = 0.19	0.79 (0.59-1.06), *P* = 0.12
Procedural outcomes						
Cardioversion	33 (1.18%)	44 (1.58%)	990 (0.01%)	46.34 (31.72-67.71), *P* < 0.001	61.60 (44.40-85.30), *P* < 0.001	1.33 (0.84-2.10), *P* = 0.22
AF/AFL ablation	36 (1.28%)	60 (2.16%)	502 (0.01%)	191.30 (136.20-268.60), *P* < 0.001	263.90 (202.00-344.60), *P* < 0.001	1.38 (1.06-1.80), *P* = 0.018
SVT ablation	30 (1.07%)	84 (3.02%)	3,831 (0.05%)	13.87 (9.59-20.07), *P* < 0.001	37.80 (30.10-47.40), *P* < 0.001	2.72 (1.79-4.14), *P* < 0.001
VT ablation	6 (0.21%)	6 (0.22%)	799 (0.01%)	6.34 (2.65-15.19), *P* < 0.001	5.09 (2.08-12.45), *P* < 0.001	0.80 (0.26-2.50), *P* = 0.71
Pacemaker implant	31 (1.11%)	57 (2.05%)	77,650 (1.10%)	1.78 (1.37-2.31), *P* < 0.001	1.18 (0.83-1.68), *P* = 0.35	0.66 (0.43-1.03), *P* = 0.06
ICD implant	14 (0.50%)	17 (0.61%)	4,195 (0.06%)	2.82 (1.62-4.89), *P* < 0.001	3.43 (1.37-8.60), *P* < 0.001	1.22 (0.60-2.47), *P* = 0.59
Fetal outcomes						
Preterm birth	20 (0.7%)	22 (0.8%)	23,174 (0.3%)	2.12 (1.37-3.28), *P* < 0.001	2.34 (1.54-3.57), *P* < 0.001	1.11 (0.60-2.05), *P* = 0.74
Still birth	26 (0.9%)	34 (1.2%)	41,072 (0.6%)	1.50 (1.02-2.19), *P* = 0.04	1.96 (1.40-2.75), *P* < 0.001	1.31 (0.78-2.18), *P* = 0.30
Small for gestational age	18 (0.6%)	17 (0.6%)	59,301 (0.8%)	0.89 (0.56-1.42), *P* = 0.62	0.84 (0.52-1.35), *P* = 0.46	0.90 (0.47-1.73), *P* = 0.76

Event counts and percentages for clinical, procedural, and fetal outcomes are presented for AF/AFL cohort. Values are n (%). Multivariable-adjusted HR from Cox proportional hazards models are reported for clinical and procedural outcomes. Multivariable-adjusted OR from logistic regression are reported for fetal outcomes for each pairwise comparison among women with new-onset AF/AFL during pregnancy, AF/AFL prior to pregnancy, and no AF/AFL.

AF = atrial fibrillation; AFL = atrial flutter; AMI = acute myocardial infarction; HF = heart failure; HR = hazard ratio; ICD = implantable cardioverter-defibrillator; MACE = major adverse cardiovascular events; SVT = supraventricular tachycardia; VT = ventricular tachycardia.

The long-term incidence of cardiac procedures was significantly higher among pregnant women with both new-onset and pre-existing AF/AFL than among arrhythmia-free controls, including cardioversion (33 [1.18%] and 44 [1.58%] vs 990 [0.01%], both *P* < 0.001), AF/AFL ablation (36 [1.28%] and 60 [2.16%] vs 502 [0.01%], both *P* < 0.001), and SVT ablation (30 [1.07%] and 84 [3.02%] vs 3,831 [0.05%], both *P* < 0.001). AF/AFL ablation and SVT ablation rates were significantly higher in women with pre-existing vs new-onset AF/AFL (both *P* < 0.001) (Table 2).

#### Fetal outcomes at delivery

Compared to arrhythmia-free controls, pregnant women with new-onset AF/AFL were significantly more likely to experience adverse fetal outcomes including preterm birth (OR: 2.12; 95% CI: 1.37-3.28; *P* < 0.001) and stillbirth (OR: 1.50; 95% CI: 1.02-2.19; *P* = 0.04) (Table 2). Similar risks were noted for women with pre-existing AF/AFL compared to arrhythmia-free controls (preterm birth OR: 2.34; 95% CI: 1.54-3.57; *P* < 0.001, and stillbirth OR: 1.96; 95% CI: 1.40-2.75; *P* < 0.001). The risk of preterm birth and still birth was similar for the new-onset and pre-existing AF/AFL groups. The risk of small-for-gestation-age babies was not significantly increased in either AF/AFL group, suggesting that AF/AFL-associated fetal risk is driven primarily by preterm delivery and fetal loss rather than by impaired intrauterine growth.

### SVT cohort

Among 6,611,658 women with pregnancies in our dataset, 12,860 (0.19%) received a new diagnosis of SVT during pregnancy, while 45,288 (0.69%) had an established diagnosis of SVT prior to pregnancy. The median post-index pregnancy follow-up duration in the SVT cohort was 6.7 years (interquartile range: 3.8-9.8). Baseline characteristics across SVT subgroups are detailed in Table 3. Both women with new-onset and those with pre-existing SVT were older and had a greater burden of comorbidities than women without SVT.

**Table 3:** Baseline Characteristics of the SVT Cohort

	New-Onset SVT (n = 12,860)	Pre-Existing SVT (n = 45,288)	No SVT (n = 6,553,510)	*P* Value
Age, y				<0.0001
<20	498 (3.9%)	2,428 (5.4%)	493,376 (7.5%)	
20-29	6,250 (48.6%)	21,633 (47.8%)	3,338,727 (50.9%)	
30-34	3,794 (29.5%)	12,906 (28.5%)	1,735,613 (26.5%)	
35-39	1,891 (14.7%)	6,627 (14.6%)	804,691 (12.3%)	
≥40	427 (3.3%)	1,695 (3.7%)	181,103 (2.8%)	
Race/ethnicity				<0.0001
Hispanic	1,324 (10.3%)	3,745 (8.3%)	1,446,848 (22.1%)	
American Indian/Alaska Native	117 (0.9%)	452 (1.0%)	58,662 (0.9%)	
Asian	461 (3.6%)	1,478 (3.3%)	400,944 (6.1%)	
Black	1,621 (12.6%)	5,678 (12.5%)	1,174,942 (17.9%)	
Native Hawaiian/Pacific Islander	46 (0.4%)	186 (0.4%)	32,672 (0.5%)	
White	9,105 (70.8%)	33,336 (73.6%)	3,303,834 (50.4%)	
Comorbidities				
Type 2 diabetes mellitus	60 (0.5%)	341 (0.8%)	20,712 (0.3%)	<0.0001
Hypertension	129 (1.0%)	957 (2.1%)	41,003 (0.6%)	<0.0001
Device implant	34 (0.3%)	112 (0.2%)	482 (0.0%)	<0.0001
Valvular heart disease	1,328 (10.3%)	2,607 (5.8%)	43,873 (0.7%)	<0.0001
Pregnancy-related comorbidities				
Gestational hypertension	700 (5.4%)	2,389 (5.3%)	215,951 (3.3%)	<0.0001
Preeclampsia and/or eclampsia	131 (1.0%)	676 (1.5%)	59,198 (0.9%)	<0.0001
Gestational diabetes	879 (6.8%)	2,711 (6.0%)	305,759 (4.7%)	<0.0001
Preterm labor	1,338 (10.4%)	3,557 (7.9%)	384,052 (5.9%)	<0.0001
Anemia during pregnancy	2,103 (16.4%)	4,126 (9.1%)	572,215 (8.7%)	<0.0001
Obesity during pregnancy	2,184 (17.0%)	4,993 (11.0%)	723,518 (11.0%)	<0.0001
Smoking during pregnancy	976 (7.6%)	3,146 (6.9%)	344,534 (5.3%)	<0.0001
Alcohol abuse	24 (0.2%)	129 (0.3%)	8,182 (0.1%)	<0.0001

Values are n (%) or median (Q1-Q3), unless otherwise indicated.

SVT = supraventricular tachycardia.

#### Long-term maternal outcomes

**Figure 3:**
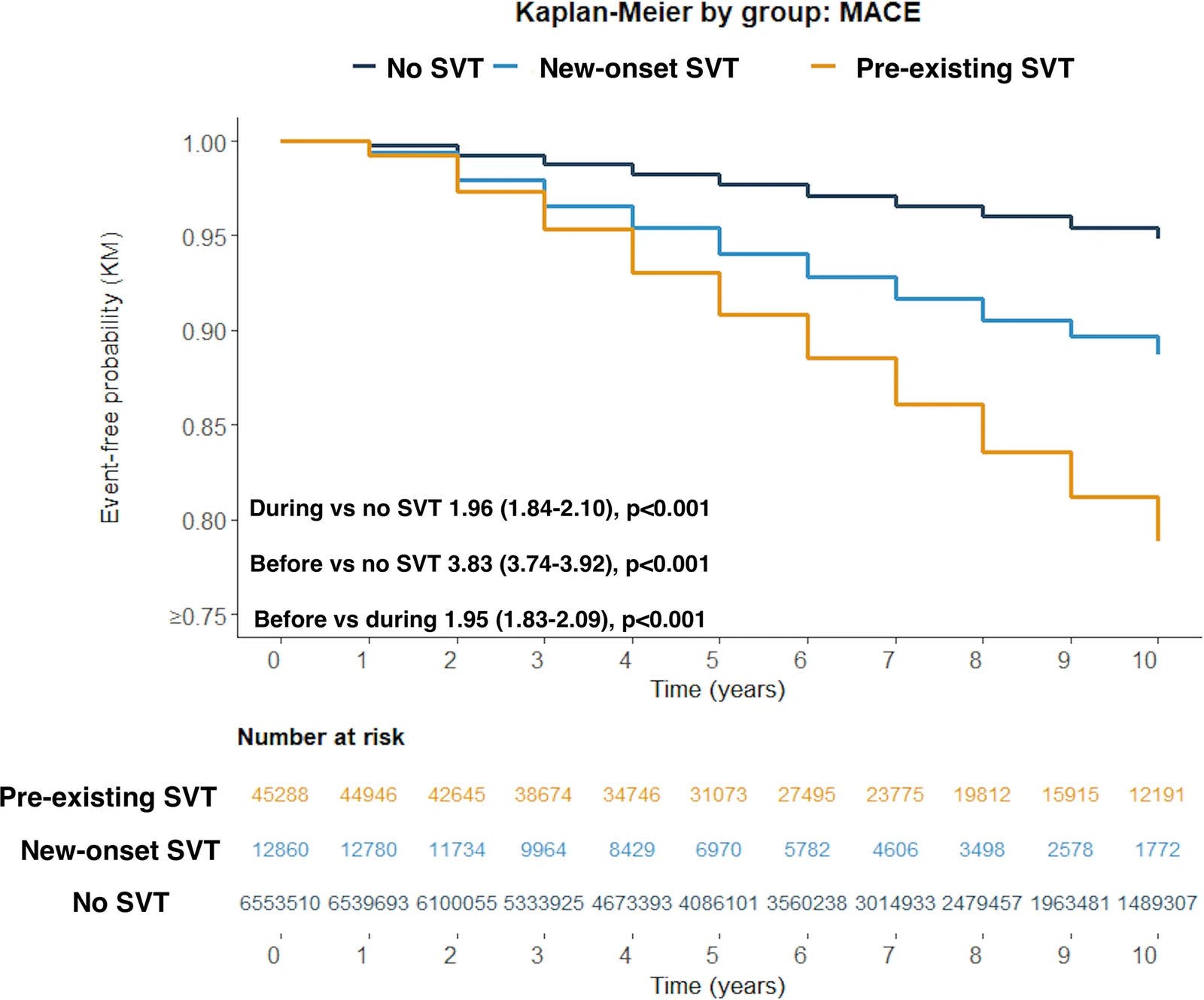
Kaplan-Meier Curves for MACE-Free Probability of SVT Cohort Kaplan-Meier curves for women with SVT during pregnancy (n = 12,860), pre-existing SVT prior to pregnancy (n = 45,288), and no SVT history (n = 6,553,510). Event-free probability of MACE is plotted from the index pregnancy encounter over 10 years of follow-up. Numbers at risk are shown below each panel at yearly intervals. MACE = major adverse cardiovascular events; SVT = supraventricular tachycardia.

Both SVT groups experienced significantly increased long-term cardiovascular risk compared to controls (Table 4, Figure 3). Pregnant women with a new diagnosis of SVT experienced significantly increased long-term risks including MACE (HR: 1.96; 95% CI: 1.84-2.10), AMI (HR: 3.43; 95% CI: 2.75-4.27), HF hospitalization (HR: 3.21; 95% CI: 2.86-3.60), stroke (HR: 1.95; 95% CI: 1.73-2.18), and major bleeding (HR: 1.58; 95% CI: 1.46-1.72), all *P* < 0.001. Similarly, pregnant women with a pre-existing diagnosis of SVT also experienced increased long-term risk of MACE, AMI, HF hospitalization, stroke, and major bleeding (HR: 3.83, 10.69, 8.91, 5.06, and 2.49, respectively, all *P* < 0.001). Direct comparisons demonstrated that women with a pre-existing SVT diagnosis were at increased long-term risk compared to women with a new SVT diagnosis during pregnancy, including MACE (HR: 1.95; 95% CI: 1.83-2.09) and HF hospitalization (HR: 2.77; 95% CI: 2.46-3.12), both *P* < 0.001. In a sensitivity analysis excluding HF hospitalization from the MACE definition (composite of AMI and stroke only), associations remained significant for both new-onset SVT (HR: 2.45; 95% CI: 2.10-2.87, *P* < 0.001) and pre-existing SVT (HR: 6.90; 95% CI: 6.60-7.22; *P* < 0.001) compared to controls (Supplemental Table 4).

**Table 4:** Event Rates and Outcomes for SVT Cohort

	Event rates			Outcomes		
	New-Onset SVT	Pre-Existing SVT	No SVT	New-Onset SVT vs No SVTHR/OR (95% CI), *P* value	Pre-Existing SVT vs No SVTHR/OR (95% CI), *P* value	Pre-Existing SVT vs New-onset SVTHR/OR (95% CI), *P* value
Clinical outcomes						
MACE	940 (7.31%)	2,713 (17.0%)	233,520 (3.56%)	1.96 (1.84-2.10), *P* < 0.001	3.83 (3.74-3.92), *P* < 0.001	1.95 (1.83-2.09), *P* < 0.001
AMI	81 (0.63%)	1,227 (2.71%)	11,682 (0.18%)	3.43 (2.75-4.27), *P* < 0.001	10.69 (10.06-11.36), *P* < 0.001	3.11 (2.49-3.91), *P* < 0.001
HF	302 (2.35%)	3,316 (7.32%)	37,346 (0.57%)	3.21 (2.86-3.60), *P* < 0.001	8.91 (8.58-9.24), *P* < 0.001	2.77 (2.46-3.12), *P* < 0.001
Stroke	85 (0.66%)	1,052 (2.32%)	21,085 (0.32%)	1.95 (1.73-2.18), *P* < 0.001	5.06 (4.76-5.39), *P* < 0.001	2.60 (2.31-2.93), *P* < 0.001
Major bleeding	551 (4.28%)	3,824 (8.44%)	176,297 (2.69%)	1.58 (1.46-1.72), *P* < 0.001	2.49 (2.41-2.57), *P* < 0.001	1.57 (1.44-1.72), *P* < 0.001
Procedural outcomes						
Cardioversion	47 (0.37%)	532 (1.17%)	600 (0.01%)	36.02 (26.62-48.73), *P* < 0.001	86.23 (76.28-97.48), *P* < 0.001	2.39 (1.77-3.23), *P* < 0.001
AF/AFL ablation	27 (0.21%)	262 (0.58%)	376 (0.01%)	34.08 (22.92-50.66), *P* < 0.001	64.30 (54.57-75.77), *P* < 0.001	1.89 (1.27-2.81), *P* = 0.002
SVT ablation	754 (5.86%)	3,338 (7.37%)	14 (0.00%)	71.6 (61.2-79.9), *P* < 0.001	78.7 (67.3-81.8), *P* < 0.001	1.21 (1.04-1.43) *P* < 0.001
VT ablation	36 (0.28%)	229 (0.51%)	578 (0.01%)	24.66 (17.45-34.84), *P* < 0.001	35.67 (30.37-41.90), *P* < 0.001	1.45 (1.02-2.06), *P* = 0.041
Pacemaker implant	194 (1.51%)	1,566 (3.46%)	76,492 (1.17%)	1.36 (1.18-1.56), *P* < 0.001	2.52 (2.40-2.65), *P* < 0.001	1.86 (1.60-2.15), *P* < 0.001
ICD implant	30 (0.23%)	429 (0.95%)	3,864 (0.06%)	3.27 (2.28-4.71), *P* < 0.001	10.59 (9.53-11.77), *P* < 0.001	3.23 (2.23-4.68), *P* < 0.001
Fetal outcomes						
Preterm birth	78 (0.6%)	203 (0.4%)	22,433 (0.3%)	1.77 (1.42-2.21), *P* < 0.001	1.30 (1.13-1.50), *P* < 0.001	0.73 (0.56-0.96), *P* = 0.02
Still birth	3 (0.0%)	19 (0.0%)	2,499 (0.0%)	0.61 (0.20-1.89), *P* = 0.40	1.10 (0.70-1.72), *P* = 0.69	1.79 (0.53-6.05), *P* = 0.35
Small for gestational age	80 (0.6%)	303 (0.7%)	54,723 (0.8%)	0.80 (0.64-1.00), *P* = 0.047	0.86 (0.77-0.96), *P* = 0.01	1.08 (0.84-1.38), *P* = 0.55

Event counts and percentages for clinical, procedural, and fetal outcomes are presented for SVT cohort. Multivariable-adjusted HR from Cox proportional hazards models are reported for clinical and procedural outcomes. Multivariable-adjusted OR from logistic regression are reported for fetal outcomes for each pairwise comparison among women with new-onset SVT during pregnancy, SVT prior to pregnancy, and no SVT.

AF = atrial fibrillation; AFL = atrial flutter; AMI = acute myocardial infarction; HF = heart failure; HR = hazard ratio; ICD = implantable cardioverter-defibrillator; MACE = major adverse cardiovascular events; SVT = supraventricular tachycardia; VT = ventricular tachycardia.

The long-term need for cardiac procedures was significantly higher in both SVT groups than in arrhythmia-free controls, including SVT ablation (754 [5.86%] and 3,338 [7.37%] vs 14 [0.00%], both *P* < 0.001), pacemaker implantation (194 [1.51%] and 1,566 [3.46%] vs 76,492 [1.17%], both *P* < 0.001), and ICD implantation (30 [0.23%] and 429 [0.95%] vs 3,864 [0.06%], both *P* < 0.001). Procedural rates were significantly higher in women with pre-existing SVT than in those with new-onset SVT across all procedure types (Table 4).

#### Fetal outcomes at delivery

SVT during pregnancy was associated with significantly elevated odds of preterm birth compared to arrhythmia-free controls (OR: 1.77; 95% CI: 1.42-2.21; *P* < 0.001). However, a new diagnosis of SVT during pregnancy was not associated with significantly increased risk of stillbirth (OR: 0.61; *P* = 0.40) or SGA (OR: 0.80; *P* = 0.047). Pregnant women with a pre-existing SVT diagnosis demonstrated increased risk of preterm birth (OR: 1.30; *P* < 0.001). On direct comparison, the risk of preterm birth was slightly higher with a new diagnosis of SVT during pregnancy than with a pre-existing SVT diagnosis (Table 4).

## Discussion

Based on a large-scale analysis encompassing over 7.6 million pregnancies from the Epic Cosmos database, this study represents, to our knowledge, the largest analysis to date of long-term maternal cardiovascular outcomes and fetal outcomes in pregnant women with AF/AFL or SVT, and the first to directly compare outcomes according to whether the arrhythmia preceded or was newly diagnosed during pregnancy. In this study, we report 4 principal findings: 1) both AF/AFL and SVT, regardless of whether they are diagnosed prior to or during pregnancy, are independently associated with significantly increased long-term maternal cardiovascular morbidity relative to arrhythmia-free controls; 2) women with new-onset AF/AFL during pregnancy face long-term cardiovascular risks statistically indistinguishable from those with pre-existing AF/AFL; 3) SVT demonstrates a risk gradient with pre-existing SVT conferring significantly increased long-term cardiovascular risk to the mother compared to SVT diagnosed during pregnancy; 4) adverse fetal outcomes, particularly preterm birth, are elevated across arrhythmia types.

The incidence of arrhythmias during pregnancy has been increasing over recent decades, driven in part by an increasing average age of childbirth among women. A number of studies have demonstrated that arrhythmias during pregnancy are associated with adverse maternal and fetal outcomes.^5^^,^^10^, ^11^, ^12^ However, prior studies have been limited to relatively short-term, primarily peripartum follow-up, thus precluding assessment of the impact of new-onset arrhythmias during pregnancy on long-term maternal cardiovascular outcomes. Our study extends prior data by demonstrating that over longer-term follow-up (median follow-up 6.7 years), both newly diagnosed AF/AFL and SVT during pregnancy are associated with significantly increased maternal cardiovascular risk compared to pregnant women without arrhythmias. These observations are consistent with underlying myocardial substrate vulnerability and shared cardiometabolic risk factors that predispose women both to arrhythmia and to long-term adverse events.^13^ A new peripartum AF/AFL or SVT diagnosis should therefore be framed as a sentinel finding that may identify women at elevated long-term cardiovascular risk who would benefit from structured follow-up.

Our data also shed light on the extent to which new-onset arrhythmia during pregnancy is a manifestation of transient and reversible hemodynamic and physiologic changes during pregnancy vs a reflection of a vulnerable underlying substrate. The hemodynamic and physiologic adaptations of pregnancy—including a 30% to 50% increase in plasma volume, elevated resting heart rate, and shifts in autonomic tone—create an electrophysiological stress that may trigger arrhythmias in women with an underlying vulnerable substrate.^3^^,^^14^ In the AF/AFL cohort, the magnitude of long-term maternal cardiovascular risk was similar among women with a new arrhythmia diagnosis during pregnancy compared to women with a pre-existing diagnosis of AF/AFL. This finding suggests that the presence of AF/AFL, irrespective of when it is first diagnosed relative to pregnancy, identifies a woman who is susceptible to long-term cardiovascular risk and that AF/AFL during pregnancy is primarily a marker of an underlying vulnerable substrate. Data from the ROPAC (Registry on Pregnancy and Cardiac Disease) registry demonstrate that AF/AFL during pregnancy in patients with structural heart disease is associated with unfavorable maternal outcomes, with pre-existing AF/AFL being the strongest predictor of arrhythmia recurrence during pregnancy.^11^

In contrast to the AF/AFL cohort, where long-term cardiovascular risk was similar between pregnant women with a new diagnosis during pregnancy vs those with a pre-existing diagnosis, in the SVT cohort, a pre-existing SVT diagnosis was associated with a 2- to 3-fold higher risk of maternal major cardiovascular adverse outcomes compared to a new SVT diagnosis during pregnancy. This finding suggests that for some women with a new diagnosis of SVT during pregnancy, the arrhythmia may be triggered by reversible hemodynamic and physiologic changes during pregnancy such that the long-term risk associated with a new diagnosis of SVT during pregnancy is lower than the risk associated with a pre-existing SVT diagnosis. Although SVT is traditionally considered a benign arrhythmia, growing evidence suggests this may not hold true in the context of pregnancy.^15^ Our group recently demonstrated that SVT during pregnancy-related hospitalization was associated with significantly increased in-hospital maternal mortality,^16^ reinforcing that pre-existing SVT in particular may identify a higher-risk population with significant underlying structural and cardiometabolic substrate. Similarly, among healthy women with structurally normal hearts, SVT during pregnancy was an independent predictor of preterm labor and cesarean delivery, reinforcing that even in low-risk populations, SVT carries clinical consequences during pregnancy.^17^ Our data complement prior studies by demonstrating the differential impact on long-term maternal outcomes of a pre-existing vs a new diagnosis of SVT during pregnancy. It is important to acknowledge that SVT encompasses a heterogeneous group of arrhythmias including atrioventricular nodal reentrant tachycardia, atrioventricular reentrant tachycardia, and atrial tachycardia, with distinct electrophysiologic mechanisms and prognoses. However, in real-world clinical practice, SVT is frequently diagnosed and managed as a unified category prior to formal electrophysiologic study characterization, and our findings therefore reflect the real-world risk profile of women presenting with SVT in the context of pregnancy.

The impact of maternal arrhythmias during pregnancy on fetal outcomes remains incompletely characterized. Although some studies have demonstrated an increased risk of adverse fetal outcomes associated with maternal AF/AFL or SVT,^18^^,^^19^ after adjustment for other risk factors, whether or not maternal arrhythmias confer an independent risk of adverse fetal outcomes is unclear. Among pregnant women with structurally normal hearts, maternal SVT during pregnancy does not seem to confer increased fetal risk in relatively small studies.^20^ The Epic Cosmos database offers a unique opportunity to evaluate the association between maternal arrhythmias and fetal outcomes by facilitating linkage between the mother’s and the baby’s electronic medical records. Our data demonstrate that both newly diagnosed AF/AFL during pregnancy and pre-existing AF/AFL are associated with an increased risk of preterm birth and stillbirth. In contrast, among pregnant women with a new diagnosis of SVT, the risk of preterm birth was higher than that among those with a pre-existing diagnosis of SVT.

### Study limitations

Several limitations warrant acknowledgment. First, as a real-world database analysis, Epic Cosmos relies on ICD-10 coding and EHR documentation, which may not be able to confirm true arrhythmia onset; women with limited prior healthcare access or subclinical disease may have had pre-existing arrhythmias first documented during pregnancy, potentially biasing comparisons between groups. ICD-10-based covariate adjustment may not fully capture comorbidity severity in women with arrhythmias; the observed HR in these groups may therefore partially reflect underlying disease substrate rather than an independent effect of the arrhythmia itself. Second, the arrhythmia burden, including episode frequency, duration, and ventricular response, could not be quantified, and antiarrhythmic drug use was not captured and may have modified outcomes. The absence of medication data limits our ability to fully characterize treatment-outcome relationships and represents a meaningful gap. Studies linking EHR data to pharmacy dispensing records would be needed to address this limitation. Despite the large overall cohort, absolute event numbers for some outcomes—particularly AMI and stroke—are modest, resulting in wide CI; these estimates should be interpreted with caution and warrant confirmation in future studies. Third, the follow-up completeness may vary across contributing health systems, and patient attrition from the health system network may introduce informative censoring. Fetal outcome rates are lower than national averages, likely reflecting incomplete mother-baby linkage inherent to the Epic Cosmos database, although relative comparisons between groups remain interpretable assuming linkage completeness does not differ by arrhythmia status. Fourth, women with known arrhythmias may receive more intensive cardiac surveillance following pregnancy, potentially increasing detection of subsequent events; in addition, the database does not allow reliable distinction between ongoing follow-up and loss to follow-up. Fifth, SVT encompasses a heterogeneous group of arrhythmias with distinct mechanisms and prognoses; our database does not permit differentiation between subtypes, which constrains mechanistic interpretation and warrants confirmation in future studies using registry data with detailed rhythm documentation. Lastly, as an observational study, these findings reflect statistical associations and cannot establish causality. Several potential confounders relevant to long-term cardiovascular risk—including socioeconomic status, insurance status, physical activity, dietary habits, family history of cardiovascular disease, and chronic kidney disease—were not available for adjustment in the current analysis. Granular structural and congenital heart disease data are not reliably captured in large EHR databases, and residual confounding from unmeasured structural substrates cannot be excluded. Residual confounding from these unmeasured variables may partially explain the associations observed.

## Conclusions

In a large-scale, real-world analysis of long-term maternal cardiovascular and fetal outcomes associated with new-onset arrhythmia during pregnancy, our data suggest that both AF/AFL and SVT, regardless of whether diagnosed prior to or during pregnancy, are associated with increased long-term maternal cardiovascular risk compared to arrhythmia-free controls. For AF/AFL, long-term cardiovascular risk was similar regardless of whether the diagnosis preceded or occurred during pregnancy. In contrast, for SVT, a pre-existing diagnosis was associated with greater long-term risk than a new diagnosis during pregnancy. Both arrhythmia types were associated with an increased risk of some fetal outcomes including preterm birth. These data have implications for postpartum surveillance strategies and patient counseling and are consistent with the potential value of integrating structured long-term cardiovascular follow-up into the care pathways of women who experience new-onset arrhythmia during pregnancy.

## Funding support and author disclosures

Dr Huang is supported by the Carlyle Fraser Heart Center cardiology research fellowship. The 10.13039/100006108National Center for Advancing Translational Sciences of the National Institutes of Health provided support under the award number UL1TR002378. The content is solely the responsibility of the authors and does not necessarily represent the official views of the 10.13039/100000002National Institutes of Health. All other authors have reported that they have no relationships relevant to the contents of this paper to disclose.
