# Supplementary material for: Fetal and Long-Term Maternal Outcomes of New-Onset Arrhythmia During Pregnancy: An Epic Cosmos Analysis
Source: JACC Adv. 2026 Aug 20;5(9):103135. doi: 10.1016/j.jacadv.2026.103135 (PMC13524550; doi:10.1016/j.jacadv.2026.103135)
Supplement: Supplementary file 1 [file mmc1.pdf]

Supplemental Table 1

Variable	ICD-10-CM codes
**Co-morbidities**	
Type 1 Diabetes Mellitus (T1DM)	E10.*
Type 2 Diabetes Mellitus (T2DM)	E11.*
Hypertension (HTN)	I10.*
Valvular heart disease	I05*, I06*, I07*, I08*, I34*, I35*, I36*, I37*
Obesity	E66.*
Cardiac implantable electronic device implant	Z950, Z95810
**OB-specific co-morbidities**	
Pre-existing hypertension complicating pregnancy	O10.*, O11.*
History of gestational hypertension	O12.*, O13.*, O16.*
History of preeclampsia or eclampsia	O14.*
History of gestational diabetes	O24.*
History of recurrent pregnancy loss	N96.*, O26.20, O26.21, O26.22, O26.23
History of preterm labor	O60.*
Anemia complicating pregnancy	O99.02, O99.03
Obesity complicating pregnancy	O99.210, O99.211, O99.212, O99.213, O99.214
Smoking complicating pregnancy	O99.330, O99.331, O99.332, O99.333, O99.334

Supplemental Table 2

Variable	ICD-10-CM codes	CPT codes	ICD-10-PCS codes
**Clinical outcomes**			

Acute myocardial infarction (AMI)	I21.01, I21.02, I21.09, I21.1, I21.19, I21.11, I21.2, I21.29, I21.3, I21.4, I21.9		
Heart failure (HF)	I50.*		
Stroke	I63.*, I65.*, I66.*		
Gastrointestinal bleeding (GIB)	K92.*		
Intracranial bleeding	I61.*		
Ventricular arrhythmia	I470, I472, I4901, I4902	-	-
Other supraventricular tachycardia (SVT)	I471	-	-
Conduction system disease	I440, I441, I442, I4430, I4439, I444, I445, I4460, I4469, I447, I450, I4510, I4519, I452, I453, I454, I455, I4589, I459	-	-
Sick sinus syndrome	I495	-	-
Cardiogenic shock	R57.0	-	-
Readmission due to AF/AFL	I48.*	-	-
Readmission due to heart failure	I50.*		
Cardiac arrest	I462, I468, I469, I46.*	-	-
**Procedure needs**			

Cardioversion		92960, 92961, 00410	5A2204Z
AF/AFL ablation		93656	
Left atrial appendage occlusion device implant		33340	02L70CK, 02L70DK, 02L70ZK, 02L73CK, 02L73DK, 02L73ZK, 02L74CK, 02L74DK, 02L74ZK
Other SVT ablation		93653	
Ventricular tachycardia (VT) ablation		93654	
Leadless pacemaker placement		33274, 0389T, 0387T, 0391T, 0390T, 0795T, 0801T 0802T, 0803T, 0804T	02HK3NZ, 02WA3NZ
PPM (pacemaker) placement. Single chamber or dual chamber		33206, 33207, 33208, 33212, 33213, 33214, 33215, 33216, 33217, 33218, 33220, 33221, 33222, 93724, 0796T, 0797T, 93279, 93288	0JH60PZ, 0JH63PZ, 0JH80PZ, 0JH83PZ, 0JH604Z, 0JH634Z, 0JH804Z, 0JH834Z, 0JH605Z, 0JH635Z, 0JH805Z, 0JH835Z, 0JH606Z, 0JH636Z, 0JH806Z, 0JH836Z,

			02HK4JZ, 02HK3JZ, 02HK0JZ, 02WA0MZ, 02WA3MZ, 02WA4MZ, 0JWT0PZ, 0JWT3PZ
ICD placement		33215, 33216, 33217, 33218, 33220, 33230, 33240, 33249, 33231, 33223, 33235, 93292, 33223	0JH608Z, 0JH638Z, 0JH808Z, 0JH838Z, 02H43KZ, 02H43MZ, 02H63KZ, 02HK3KZ, 02HN0KZ, 02HN4KZ, 02HK4KZ, 02HK0KZ, 02H44KZ, 02H60KZ, 02H64KZ
Subcutaneous or substernal ICD		33271, 33272, 0319T, 0320T, 0325T, 0326T, 0327T, 0328T, 93261, 93644, 0572T	0JH639Z
CRT placement		33221, 33224, 33225, 33226, 33229, 33263, 33264	02H40JZ, 02H43JZ, 02H44JZ, 02HL0JZ, 02HL3JZ, 02HL4JZ, 0JH607Z, 0JH637Z, 0JH807Z, 0JH837Z
**Fetal outcomes**			
Preterm birth	P07.*	-	-

Small for gestational age	P05.*	-	-
Stillbirth	P95.*<br>Z37.1<br>Z37.4<br>Z37.7<br>Z37.3	-	-

Supplemental Table 3

Outcome	Event rates			Adjusted HR (95% CI), p-value		
	AF/AFL during pregnancy	No AF/AFL	AF/AFL before pregnancy	AF/AFL during pregnancy vs No AF/AFL	AF/AFL before pregnancy vs No AF/AFL	AF/AFL before pregnancy vs AF/AFL during pregnancy
MACE no HF	51 (1.84%)	32,145 (0.45%)	50 (1.78%)	2.88 (2.18-3.80) p<0.001	3.09 (2.34-4.10) p<0.001	1.08 (0.73-1.59) p=0.716

Supplemental Table 4

Outcome	Event rates			Adjusted HR (95% CI), p-value		
	SVT during pregnancy	No SVT	SVT before pregnancy	SVT during	SVT before	SVT before pregnancy

				pregnancy vs No SVT	pregnancy vs No SVT	vs SVT during pregnancy
MACE no HF	161 (1.25%)	32,015 (0.49%)	2,144 (4.73%)	2.45 (2.10-2.87) p<0.001	6.90 (6.60-7.22) p<0.001	2.81 (2.40-3.30) p<0.001
